# A Real-World Data Derived Pharmacovigilance Assessment on Drug-Induced Nephropathy: Implication on Gaps in Patient Care

**DOI:** 10.3390/healthcare12010095

**Published:** 2023-12-31

**Authors:** Yujin Kim, Chang-Young Choi, Yongjun Sunwoo, Chaerin Go, Semi Kim, Sae Hyun Eom, Sooyoung Shin, Yeo Jin Choi

**Affiliations:** 1 Department of Regulatory Science, Graduate School, Kyung Hee University, Seoul 02447, Republic of Korea; yuriyuri@khu.ac.kr; 2 Department of Internal Medicine, Ajou University Medical Center, Suwon 16499, Republic of Korea; changchoi1216@gmail.com; 3 Department of Pharmacy, College of Pharmacy, Kyung Hee University, Seoul 02447, Republic of Korea; dydwns3567@khu.ac.kr (Y.S.); chrin07@khu.ac.kr (C.G.); sarah1432@khu.ac.kr (S.K.); djachl01@khu.ac.kr (S.H.E.); 4 Department of Pharmacy, College of Pharmacy, Ajou University, Suwon 16499, Republic of Korea; 5 Research Institute of Pharmaceutical Science and Technology (RIPST), Ajou University, Suwon 16499, Republic of Korea; 6 Institute of Regulatory Innovation through Science (IRIS), Kyung Hee University, Seoul 02447, Republic of Korea

**Keywords:** antibiotics, drug safety, gaps in patient care, immunosuppressants, nephrotoxicity, pharmacovigilance, KAERS DB, real-world data

## Abstract

This retrospective cross-sectional study aims to investigate the prevalence and seriousness of drug-induced nephrotoxicity and to identify clinical predictors intensifying the seriousness of nephrotoxicity. Adverse drug events (ADEs) reported to the Korean Adverse Event Reporting System Database (KAERS DB) from January 2012 to December 2021 were investigated. The association between the seriousness and the etiologic drug was estimated in reporting odds ratio (ROR) based on disproportionality analysis. Logistic regression was utilized to recognize predictors associated with serious nephrotoxicity. The majority of ADEs were reported in ages 30 to 59, and immunosuppressants were the most etiologic medications. ADEs involving antibiotics, including vancomycin (ROR 0.268; 95% CI 0.129–0.557), were less likely to be serious. More than 93% of cyclosporine-related ADEs were serious nephrotoxicity, whereas tacrolimus was less likely to report serious nephrotoxicity (ROR 0.356; 95% CI 0.187–0.680). The risk of serious nephrotoxicity was decreased with aging (ROR 0.955; 95% CI 0.940–0.972) while increased in women (OR 2.700; 95% CI 1.450–5.008). Polypharmacy was associated with increased risk of interstitial nephritis (OR 1.019; 95% CI 1.001–1.038). However, further studies investigating the impact of clinical practice on ADE incidences as well as clinical prognosis related to nephrotoxicity are obligated.

## 1. Introduction

Pharmacotherapy is considered a fundamental treatment modality in most acute and chronic disorders, including cardiovascular, endocrinological, psychiatric, infectious, and even autoimmune disorders. As the incidences of chronic disorders such as hypertension, dyslipidemia, diabetes mellitus (DM), and cancer generally increase with age, the prevalence of multimorbidity tends to escalate with aging, leading to an increase in the number of concomitant medications [1,2]. Recent studies have indeed demonstrated a multimorbidity prevalence of 62% and 81.5% in patients aged 65 to 74 years and 85 years or older, respectively, implying a continuous increase in drug exposure in aged populations [3]. Moreover, considering that the elderly patients are more vulnerable to acute communicable diseases, such as pneumonia, secondary to underlying comorbidities and declined immune functions, the number of concomitant medications as well as drug exposures may be greater than estimated [4].

Polypharmacy, characterized by daily administration of at least five concomitant medications, has become a significant healthcare issue worldwide, especially in elderly populations [5]. Polypharmacy is associated with substantial adverse clinical prognoses such as increased mortality, falls, drug interactions, and adverse drug events (ADEs) [6]. Hence, decreasing inappropriate drug use as well as providing optimal pharmacotherapy are warranted to promote patient safety and improve clinical prognoses. Nevertheless, myriad factors including genetics, pharmacokinetic and pharmacodynamic characteristics, polypharmacy, and comorbidities may play critical roles as determinants of altered responses to the medications, consequently increasing the risk of ADEs [7]. The ADE incidences, including serious adverse events (SAEs), remain high, especially in hospitalized patients, despite substantial under-reporting of ADEs in real clinical practice [8]. Moreover, the majority of preventable ADEs are associated with polypharmacy, drug interactions, and multimorbidity, accounting for at least 19 to 32.3% of all ADE cases, highlighting the clinical significance of gaps in patient care [9], defined as a discrepancy between recommended evidence-based care and the care that is actually provided to the patient [9,10,11,12].

The kidney is an essential organ responsible for xenobiotic excretion including medications, electrolyte and fluid homoeostasis, erythropoietin production, and vitamin D metabolism [13]. With a large number of medications being excreted renally, the kidneys are more prone to developing medication-related toxicity [14]. Drug-induced nephrotoxicity, primarily manifested as rapid deterioration of renal functions [15,16], subsequently induces numerous functional impairments, including glomerular or tubular dysfunction, blood pressure control, or renal endocrine function [17]. The apparent incidence of drug-induced nephrotoxicity is usually underestimated due to ambiguous causality and the broad clinical spectrums associated with nephrotoxicity [18], and this may contribute to gaps in patient care. Studies suggest the estimated prevalence of drug-induced acute kidney injury (AKI) is 14 to 66% of all AKI cases, indicating the significant clinical impact of drug-induced nephrotoxicity associated with pharmacotherapy [18].

Numerous medications such as antibiotics, nonsteroidal anti-inflammatory drugs (NSAIDs), and chemotherapeutic agents are known to be highly nephrotoxic [14]. Moreover, previous studies recognized old age, primarily 60 or older, underlying renal insufficiency, volume depletion, and comorbidities, including DM, heart failure, and sepsis as potential risk factors associated with drug-induced nephrotoxicity [19]. Nevertheless, pharmacovigilance studies utilizing real-world data (RWD) on drug-induced nephrotoxicity are lacking despite substantial factors associated with ADEs along with the broad clinical spectrum of drug-induced nephrotoxicity [20,21,22]. This gap leaves clinical significance as well as seriousness of these ADEs in clinical practice unanswered. Thus, this study aimed to assess the prevalence and seriousness of drug-induced nephrotoxicity, identify etiologic medications associated with nephrotoxicity, and investigate clinical predictors that may amplify the seriousness of nephrotoxicity using a nationwide voluntary ADE reporting system to convey clinical evidence on drug safety.

## 2. Materials and Methods

### 2.1. Data Source and Definition

This study was conducted in accordance with Strengthening the Reporting of Observational Studies in Epidemiology (STROBE) guideline [23]. This is a cross-sectional study analyzing the risk of drug-induced nephrotoxicity using ADE records spontaneously reported to the Korean Institute of Drug Safety & Risk Management (KIDS)—Korea Adverse Event Reporting System Database (KAERS DB), a nationwide voluntary spontaneous ADE reporting system available to the public and healthcare professionals including doctors, pharmacists, and nurses, from January 2012 to December 2021. All ADE cases reported to the KAERS DB undergo causality assessment, which are further verified by the healthcare professionals appointed by the Korean Institute of Drug Safety & Risk Management (Mistry of Food and Drug Safety). This verification is based on patients’ medical history and charts, interviews with patient and healthcare professionals, and scientific pharmacovigilance data from manufacturers to minimize biases [24,25]. All ADEs were reported using either included terms or preferred terms in accordance with the Medical Dictionary for Regulatory Activities (MedDRA) terminology, an international medical terminology used to describe ADEs [26], and ADEs were further categorized into system organ class (SOC). All ADE reports related to drug-induced nephrotoxicity [27], primarily reported as urinary system disorder per SOC, were collected from KAERS DB. The prespecified MedDRA terminology for drug-induced nephrotoxicity is summarized in Table 1. MedDRA terminology for “toxic nephropathy” refers to any adverse functional or structural changes in the kidney secondary to the effect of a chemical, including medication [28]. ADE records with “certain”, “probable/likely”, and “possible” causality per the World Health Organization-Uppsala Monitoring Centre (WHO-UMC) criteria were included in the analysis [29]. Any irrelevant ADE cases as well as masked etiologic medications (MSK coded) were excluded from the analysis. Irrelevant ADE cases were defined as any ADE reports with WHO-UMC causality of “unlikely”, “conditional/unclassified”, or “unassessable/unclassifiable” [29]. KAERS DB assigns an MSK code to medication products that have been marketed by a single or two pharmaceutical companies. The following information was extracted from the KAERS DB for the analysis: (1) patient demographic information including age and sex; (2) medical history including indication and comorbidity; (3) information of medication administrations; (4) ADE information with causality results; and (5) seriousness of ADEs. SAE were classified by the International Conference on Harmonization (ICH) E2D guideline: death, life-threatening conditions, persistent or significant disability or incapacity, hospitalization or prolongation of existing hospitalization, congenital abnormalities or birth defects, and other medically significant events [30]. The study protocol for utilizing the KAERS DB database was approved by the Korea Institute of Drug Safety & Risk Management (No. 2301A0004) (Ministry of Food and Drug Safety) and the institutional review board (IRB) of Kyung Hee University (NO. KHSIRB-23-133) (Seoul, South Korea), and informed consents were exempted by the board.

### 2.2. Statistical Analysis

SPSS Statistics 26.0 (IBM SPSS Statistics for Windows, Armonk, NY, USA) was utilized for all statistical analyses. Descriptive statistic methods were applied to summarized patient demographics and ADE frequency. Continuous variables such as age were expressed as median (range) based on the results of Kolmogorov–Smirnov normality test. The association of seriousness of drug-induced nephrotoxicity and causative drugs or drug classes was estimated using disproportionality analysis and was expressed in reporting odds ratios (RORs) with corresponding 95% confidence intervals (CIs) and Mantel–Haenszel adjusted *p*-values. RORs were estimated for specific offending medications or drug classes with at least three SAE reports [31,32]. Univariate analysis was conducted to identify predictors associated with SAEs and certain types of drug-induced nephrotoxicity, and factors included age, causality, sex, and polypharmacy (concurrent administration of at least 5 daily medications). Multiple logistic regression analysis with an enter method was utilized to identify predictors associated with risk of certain types of drug-induced nephrotoxicity as well as SAE risks including overall SAEs and hospitalizations based on the univariate logistic regression. Clinical plausibility was considered for all statistical analyses. Any *p*-values < 0.05 were considered statistically significant.

## 3. Results

### 3.1. Baseline Demographics

Among 21,081 ADE reports obtained by the Korean Institute of Drug Safety& Risk Management (Ministry of Food and Drug Safety) from January 2012 to December 2021, 304 drug-induced nephrotoxic ADE reports were included in the analysis after excluding ADE cases with irrelevant causality or MSK coded medications. The data extraction was performed in February 2023 from the database. The baseline characteristics of patients included in the analysis are described in Table 2. More than 60% of patients were men. The most ADE cases were reported in patients aged 50 to 59 (*n* = 53; 24.4%), followed by the age group 60 to 69 (*n* = 31; 14.3%). The most commonly reported types of drug-induced nephrotoxicity were toxic nephropathy (*n* = 90; 29.6%), followed by kidney failure (acute and chronic) (*n* = 72; 23.7%) and interstitial nephritis (*n* = 45; 14.8%). The number of SAE reports was higher than nonserious ADE reports, and the majority of ADE cases were reported by doctors and pharmacists (*n* = 239; 78.6% and *n* = 42; 13.8%, respectively). A total of 143 patients (47.0%) reported only 1 medication, and 28 patients (9.2%) reported at least 5 concurrent medications, otherwise referred to as polypharmacy. Infection was the most prevalent comorbidity (*n* = 101; 33.2%), followed by cancer (*n* = 38; 12.5%), cardiovascular disease (*n* = 16; 5.3%), and transplantation (*n* = 16; 5.3%).

### 3.2. Etiologic Medications Implicated in Seriousness of ADEs

**Figure 1:**
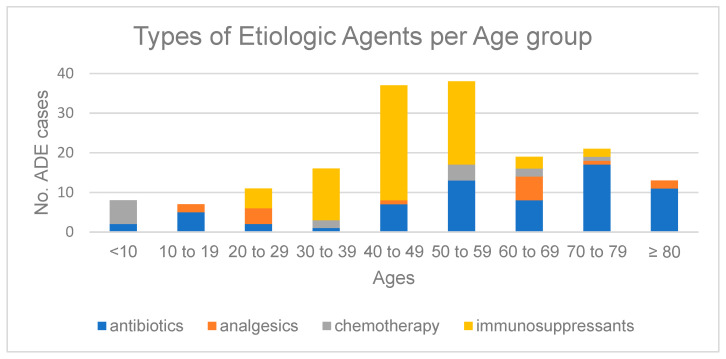
Types of etiologic agents per age group.

The medication class most associated with drug-induced nephrotoxicity was immunosuppressants (*n* = 79; 26.0%), followed by glucocorticoids (*n* = 73; 24.0%) and antibiotics (*n* = 69; 22.7%) (Table 3). The most etiologic agents for nephrotoxicity were dexamethasone (*n* = 72; 23.7%) and tacrolimus (*n* = 49; 16.1%), where all ADE reports associated with dexamethasone were SAE. ADE cases induced by immunosuppressants, including cyclosporine, tacrolimus, and everolimus, were frequently reported in patients ages between 40 and 59, whereas antibiotic-induced ADE cases were more prevalent in elderly patients ages greater than 70 (Figure 1). The association between etiologic medication case and seriousness of ADEs is summarized in Table 4. Antibiotics and antimycobacterial agents were less likely to report SAEs (ROR 0.24; 95% CI 0.133–0.433, *p* < 0.001), especially vancomycin (ROR 0.268; 95% CI 0.120–0.557; *p* < 0.001), whereas other common etiologic medications such as analgesics, chemotherapy agents, and immunosuppressants seem to have an insignificant risk of reporting SAEs (Table 4). However, tacrolimus-related nephrotoxic ADE cases were less likely to be associated with serious nephrotoxic ADEs (ROR 0.356; 95% CI 0.187–0.680, *p* = 0.002).

### 3.3. Predictors Associated with Increased Risk of Drug-Induced Nephrotoxic ADEs

The logistic analysis demonstrated significance associated with drug-induced nephrotoxicity with sex, age, and polypharmacy (Table 5). Aging was the significant predictor for increased odds of reports of interstitial nephritis and SAEs including hospitalization (Table 5); aging increased the odds of reporting interstitial nephritis (OR 1.019; 95% CI 1.001–1.038; *p =* 0.041). However, the risks of reporting overall serious drug-induced nephrotoxicity (OR: 0.955; 95% CI 0.940–0.972; *p* < 0.001), including hospitalization or prolonged hospitalization (OR 0.968; 95% CI 0.952–0.984; *p* < 0.001), were substantially lower with aging (Table 5). Women were more likely to have increased risks of reporting SAEs including mortality, hospitalization, life-threatening conditions, and disability (OR 2.700; 95% CI 1.450–5.008; *p =* 0.002). On the other hand, polypharmacy increased the risk of reporting drug-induced interstitial nephritis (OR 4.190; 95% CI 1.768–9.926; *p* = 0.001) while decreasing the risk associated with toxic nephrotoxicity (OR 0.054; 95% CI 0.007–0.408; *p* = 0.005).

## 4. Discussion

Most cases of drug-induced nephrotoxicity were associated with immunosuppressants (26.0%), followed by glucocorticoids (24.0%) and antibiotics (22.7%). The prevalence of serious drug-induced nephrotoxicity was 53.6%. The primary causative agents for drug-induced nephrotoxicity were dexamethasone and tacrolimus, where all ADE reports related to dexamethasone were SAEs. However, antibiotics and antimycobacterial agents, including vancomycin, were less likely to report serious nephrotoxicity. In the cases of immunosuppressants, cyclosporine was more likely to result in severe drug-induced nephrotoxicity than tacrolimus. The majority of ADE cases reported by immunosuppressants were from patients aged between 40 and 59, while ADE cases involving antibiotics were reported more frequently in elderly patients aged over 70. Polypharmacy is one of the predictors that can substantially increase the risk of interstitial nephritis; however, the risk of toxic nephrotoxicity was substantially reduced with polypharmacy. Female sex is one of the predictors that can substantially increase the risk and severity of nephrotoxicity. On the other hand, decreasing age is the major contributing predictor associated with serious drug-induced nephrotoxicity, including ADE cases related to hospitalizations, whereas aging significantly increased the risk of interstitial nephritis.

Many antibiotics and antimycobacterial drugs are known to be nephrotoxic agents [14]. The clinical spectrum of renal toxicity induced by antibacterial agents ranges from acute minor tubular damage to severe tubular necrosis and cell death [33]. In this study, approximately 22.7% of nephrotoxic ADE cases were related to antibiotics, and vancomycin was the most etiologic antibiotic agent with 41 nephrotoxic ADE cases, including 11 SAE cases. Among variable antibiotic agents, vancomycin as well as aminoglycosides are well known for drug-induced nephrotoxicity. However, this study revealed that the risk of reporting nephrotoxic SAEs such as mortality and hospitalization or prolonged hospitalization was substantially lower with antibiotics, including vancomycin, despite the high numbers of reported ADE cases. This finding may be correlated with successful therapeutic drug monitoring (TDM) programs endorsed in clinical settings [34]. Appropriate TDM, as well as optimal antibiotic dosing guidelines, have substantially reduced the number of antibiotic-induced nephrotoxic ADEs [35,36] including serious cases. Previous studies indeed demonstrated a higher incidence of mild vancomycin-induced nephrotoxicity cases over serious events after implementing TDM in institutions [37]. Nonetheless, the majority of antibiotic-related nephrotoxicity cases were reported in the elderly populations who were already predisposed to an elevated risk of infectious diseases and ADEs secondary to polypharmacy, as well as declined renal function. Thus, further studies on risk stratification, in addition to optimal TDM guidelines geared towards geriatric patients, are warranted to improve clinical prognoses in the elderly populations.

On the contrary, immunosuppressants showed a statistically insignificant association between drug class and the seriousness of ADE despite the highest number of nephrotoxic ADE cases. However, cyclosporine is more likely to report serious nephrotoxicity when compared to tacrolimus despite the relatively low number of cyclosporine-related nephrotoxicity. Despite significant potential to induce nephrotoxicity, calcineurin inhibitors (CNIs) such as cyclosporine and tacrolimus are considered first-line treatments for preventing graft rejection after organ transplantation, including renal transplantation. Moreover, the Kidney Disease Improvement Global Outcomes (KDIGO) guideline recommends tacrolimus over cyclosporine, primarily in renal transplantation patients, due to significantly lower risk of nephrotoxicity [38]. Furthermore, meta-analyses have suggested better cost-effectiveness of tacrolimus in relation to an improved graft loss rate as well as a lower incidence of ADEs including nephrotoxicity [39,40], and this study may reinforce potentially reduced incidence of serious nephrotoxicity with tacrolimus. Additionally, potential drug–drug interactions may have potentiated the risk of serious nephrotoxicity from cyclosporine use. Both cyclosporine and tacrolimus are metabolized by the cytochrome P450 enzymes (CYP), primarily CYP3A4; however, cyclosporine tends to interact with a wider range of medications, including anti-infective agents and anti-inflammatory drugs [41]. Moreover, UDP-glucuronosyltransferase (UGTs) and transporters, including organic anion transporting polypeptides (OATPs), P-glycoproteins (P-gp), and multidrug resistance-associated protein (MPR2), may also have contributed to potential drug–drug interactions of cyclosporine with rifampin, statins, and calcium channel blockers [42,43,44]. Nonetheless, considering that the number of tacrolimus-related ADE cases is greater than cyclosporine, further studies on definitive clinical impact on seriousness of nephrotoxicity of each immunosuppressant are required. Additionally, optimal monitoring as well as prophylactic parameters related to nephrotoxicity, including the clinical impact of drug–drug interactions, are required, as most immunosuppressant-induced ADE cases were reported in patients aged 30 to 60 years.

Polypharmacy, defined as concurrent daily administration of at least five medications, is another potential contributor for a substantial increased risk of ADEs and poor clinical prognoses, including mortality, falls, and drug interactions [6]. The multivariate logistic regression reported a substantially elevated risk of drug-induced interstitial nephritis with polypharmacy. Although this study was not able to determine the clinical impact of drug–drug interaction on the potential drug-induced nephrotoxicity, polypharmacy may have indeed contributed to drug-drug interactions, consequently inducing drug-induced nephrotoxicity, as the majority of medications are metabolized and distributed by CYP450 and transporters [42,43]. Moreover, numerous clinical studies suggested polypharmacy as an independent contributor to increased ADE risks [45]. However, caution should be exercised when interpreting the data because the risk of toxic nephropathy was substantially lowered with polypharmacy, and this may be due to potential under-reporting, resulting in the relatively small number of patients on polypharmacy, approximately 9%. Hence, further investigation into the impact of polypharmacy and drug–drug interaction on potential ADEs is necessary.

The most intriguing finding of this study is associated with the highest prevalence of SAEs from dexamethasone use. Dexamethasone, a synthetic steroid with anti-inflammatory, antiallergic, and immunosuppressive properties, has been in clinical use for many years [46]. However, the risk of dexamethasone-induced nephrotoxicity is substantially low [14]. In this study, the majority of patients who reported serious dexamethasone-induced nephrotoxicity were diagnosed with multiple myeloma and other chronic disorders that may induce nephropathy such as DM and hypertension. Multiple myeloma is the second most common hematological malignancy after lymphoma, and accounts for 1% of all cancers [47]. In this study, dexamethasone-induced nephrotoxicity may be contributed by disease-specific pathology, as renal failure is fairly common in multiple myeloma patients [48]. The concomitant administration of dexamethasone and novel agents prescribed for multiple myeloma, such as thalidomide or bortezomib, usually increases the reversibility rate of renal failure within less than a year after treatment [48,49]. Nonetheless, considering that multiple myeloma being commonly diagnosed in elderly patients who are already experiencing renal insufficiency and polypharmacy, the contribution of aging as well as altered medication response should not be neglected in dexamethasone-induced nephrotoxicity. Furthermore, dexamethasone-related nephrotoxicity in patients with chronic disorders should be re-evaluated for the severity and the pathology to establish optimal guidelines for safe dexamethasone use.

The multivariate analysis demonstrated that female sex, aging, and polypharmacy are independent risk factors for increasing seriousness of drug-induced nephrotoxicity. Consistent with previous findings, women are at elevated risk of developing drug-induced nephrotoxicity, especially serious nephrotoxicity [50]. However, despite substantial contributing impact of female sex to the risk of nephrotoxicity, treatment and monitoring guidelines geared towards sex-related risks are lacking [16]. On the contrary, the most distinctive finding of this study was a substantially decreased risk of serious nephrotoxicity including those requiring hospitalizations, with aging. Although the rationale is not evident, this finding may reflect a gap in patient care closely associated with current clinical practice. Evidence has suggested a substantially increased risk of hospitalizations as well as emergency room visits secondary to ADEs in the elderly populations [51,52]. Clinicians tend to pay closer attention to the elderly population while dosing medications, as aging not only alters pharmacokinetic and pharmacodynamic characteristics, but also involves multiple comorbidities and polypharmacy, which subsequently increases the risk of ADEs. Practically, healthcare providers should aware of potential ADEs as well as drug–drug interaction regardless of the patient’s age. However, limited resources, especially limited time to care for patients, may have resulted in prioritizing patients who need more focused care, primarily elderly patients, subsequently leaving young adults with relatively limited attention [53]. Aging usually make patients vulnerable to renal toxicity, but it does not indicate that younger patients with normal renal function are safe from ADEs. In this study, the majority of ADEs reported from young adults aged less than 65 years were associated with immunosuppressants, implying that young adults are also predisposed to the risk of drug-induced nephrotoxicity regardless of age. Therefore, further studies investigating the gap in patient care on ADE incidences in regard to the clinical practice in young adults are recommended to improve clinical prognoses in these patients. Meanwhile, healthcare providers should be aware of potential drug-induced nephrotoxicity in younger adults, and perform risk stratification for every patient regardless of age.

The greatest benefit of pharmacovigilance, a surveillance on detection, assessment, and prevention of ADEs, involves improvement of the healthcare system and clinical outcomes [54], and RWD-derived pharmacovigilance assessments are suitable for detecting ADEs associated with long-term drug use as well as providing a reflection on current clinical practice [54]. Until now, a comprehensive RWD-derived pharmacovigilance on drug-induced nephrotoxicity was limited, despite its substantial clinical impact on pharmacotherapy. The valuable aspects of this study include determination of clinical significance on nephrotoxicity induced by immunosuppressants as well as glucocorticoids, particularly dexamethasone, in addition to conveying implications on potential gaps in patient care associated with current clinical practice in young adults who have a relatively low number of underlying diseases, including renal dysfunction, over elderly patients. Nonetheless, this study has several limitations to be acknowledged. First, with the Korean Institute of Drug Safety & Risk Management (KIDS)—Korea Adverse Event Reporting System Database (KAERS DB) being a voluntary ADE reporting pharmacovigilance system, cautious interpretation of the study results is required because of potential under-reporting patterns. As we have stated in the introduction, drug-induced nephrotoxicity has broad clinical spectrums as well as clinical presentations from acute to chronic renal disorders, thereby making causality obscure. Moreover, limited information regarding indications, concurrent medication therapy, and comorbidities along with MSK coded medications may hinder the reliability of this study’s results. Additionally, the interpreted results may not be generalized to determine apparent drug-induced nephrotoxicity due to the nature of the study design and small sample size, and the KAERS DB is limited to investigate the actual incidence, prevalence, associations, or seriousness of ADEs. Moreover, this study was not able to detect the clinical significance of drug–drug interactions, including herbal interactions, as well as underlying renal insufficiency on drug-induced nephrotoxicity. However, considering that KAERS DB is a nationwide spontaneous pharmacovigilance system, the number of ADE cases included in this study were estimated to be greater than the institution-based pharmacovigilance study. Moreover, considering that almost all reported ADEs were investigated by medical professionals appointed by the Korean Institute of Drug Safety & Risk Management (Ministry of Food & Drug Safety) in regards to accuracy, causality, and reporting bias, inter-reporter variability may be trivial, subsequently enhancing the validity of the results. Nonetheless, large-scaled pharmacovigilance investigations are strongly required to elaborate clinical significance of nephrotoxicity induced by other nephrotoxic medications and to endorse clinical practice that narrows gaps in patient care. Moreover, integration of RWD databases including KAERS DB, National Health Insurance Claims data, and electronic medical health data from multiple institutions may provide more outright clinical evidence on the ADE landscape associated with these drugs. 

## 5. Conclusions

The majority of drug-induced nephrotoxicity cases reported to KAERS DB, a nationwide pharmacovigilance reporting system, were reported by immunosuppressants, primarily tacrolimus. The prevalence of serious drug-induced nephrotoxicity was 53.6%, and the number of serious ADE cases was the highest with dexamethasone use, followed by tacrolimus and cyclosporine. However, cyclosporine was more likely to report severe drug-induced nephrotoxicity than tacrolimus. Antibiotics and antimycobacterial agents including vancomycin were less likely to report serious nephrotoxicity. The majority of ADE cases reported in patients aged between 30 and 59 were associated with immunosuppressants, whereas the percentage of antibiotic-induced nephrotoxicity was higher than the immunosuppressants in elderly patients aged greater than 70. Female sex is one of the crucial predictors that substantially increases the risk of serious drug-induced nephrotoxicity. Polypharmacy and aging substantially increased the risk of interstitial nephritis. However, the risk of serious drug-induced nephrotoxicity is significantly reduced in elderly patients, and this may imply gaps in patient care manifested as relative low awareness of the risk of ADEs in young adults. Nonetheless, cautious interpretation and generalization of results are required due to the small number of ADE cases. Hence, further large-scaled studies are warranted to scrutinize the impact of current clinical practice on ADE incidences in adult patients to promote patient safety. In the meantime, the use of simultaneous pharmacovigilance monitoring practices is required.

## Figures and Tables

**Table 1:** MedDRA terminology for drug-induced nephrotoxicity.

MedDRA Terminology for Drug-Induced Nephrotoxicity	MedDRA Terminology Related to Renal Function Abnormality
acute interstitial nephritis (AIN)	creatine renal clearance abnormal
acute nephritis	creatine renal clearance decreased
acute tubular necrosis (ATN)	creatinine renal clearance increased
chronic interstitial nephritis	creatine renal clearance low
disorder kidney	renal clearances low
disorder renal	renal clearance decreased
glomerulonephritis	
kidney tubular necrosis	
nephritic syndrome	
nephritis	
nephrosclerosis	
nephrotic syndrome	
nephrotic syndrome worsened	
nephrotoxicity	
nephropathy toxic	
nephrosis	
renal hypoperfusion	
renal interstitial disorder	
syndrome nephrotic	
toxic nephropathy	
toxic renal	

**Table 2:** Baseline demographics.

Sex ^a^	*n* (%)
Male	206 (67.8%)
Female	97 (31.9%)
Age ^b^	55 (0–88)
<10	9 (3.0%)
10 to 19	9 (4.6%)
20 to 29	11 (5.1%)
30 to 39	20 (9.2%)
40 to 49	40 (18.4%)
50 to 59	53 (24.4%)
60 to 69	31 (14.3%)
70 to 79	30 (13.8%)
≥80	16 (7.4%)
Causality	
Certain	5 (1.6%)
Probable/Likely	120 (39.5%)
Possible	179 (58.9%)
ADE types	
Nonserious	141 (46.4%)
Serious	163 (53.6%)
Concomitant Medications	
1	143 (47.0%)
2	105 (34.5%)
3	17 (5.6%)
4	11 (3.6%)
≥5 (polypharmacy)	28 (9.2%)
Nephrotoxicity types	
Toxic nephropathy	90 (29.6%)
Nephritis	17 (5.6%)
Nephrosis	18 (5.9%)
Interstitial nephritis	45 (14.8%)
Kidney failure (acute and chronic)	72 (23.7%)
Nephrotic syndrome	18 (5.9%)
Reduced renal function	27 (8.9%)
Others	17(5.6%)
Reporter Types ^c^	
Doctors	239 (78.6%)
Pharmacist	42 (13.8%)
Nurses	7 (2.3%)
Others	6 (2.0%)
Comorbidities ^d^	
Cancer	38 (12.5%)
Multiple myeloma	12 (31.6%)
Gastrointestinal tract	9 (23.7%)
Central nervous system	8 (21.1%)
Breast	2 (5.3%)
Non-Hodgkin’s lymphoma	2 (5.3%)
Skin/Cutaneous	2 (5.3%)
Lung	1 (2.6%)
NOS (not otherwise specified)	2 (5.3%)
Skin disorders	10 (3.3%)
Infection	101 (33.2%)
Transplantation	16 (5.3%)
Cardiovascular disease	16 (5.3%)
Gastrointestinal disease	11 (3.6%)
Musculoskeletal disease	11 (3.6%)
Renal disease	11 (3.6%)
Pain	3 (1.0%)
Pulmonary disease	2 (0.7%)
Others	17 (5.6%)

This table elaborates demographic information of ADE reports included in the analysis. Serious adverse events were classified based on the International Conference on Harmonization (ICH) E2D guideline and included any ADE reports involving death, life-threatening conditions, persistent or significantly disability or incapacity, hospitalization, or prolongation of existing hospitalization, congenital abnormalities or birth defects, and other medically significant events. ^a^ Missing 1 case (0.3%); ^b^ reported in median (range); missing 85 cases (28.0%); ^c^ missing 10 cases (3.3%); ^d^ missing 68 cases (22.4%).

**Table 3:** Etiologic agents of drug-induced nephrotoxicity.

Drug Class	Non-SAE (n = 141)	SAE (n = 163)	Total (n = 304)
Antibiotics	50 (35.7%)	19 (11.7%)	69 (22.7%)
Amikacin	1 (0.7%)	1 (0.6%)	2 (0.7%)
Amoxicillin	1 (0.7%)	1 (0.6%)	2 (0.7%)
Aztreonam	1 (0.7%)	0 (0.0%)	1 (0.3%)
Cefazolin Sodium	1 (0.7%)	0 (0.0%)	1 (0.3%)
Cefradine	1 (0.7%)	0 (0.0%)	1 (0.3%)
Ceftriaxone Sodium Hydrate	2 (1.4%)	0 (0.0%)	2 (0.7%)
Ciprofloxacin	4 (2.8%)	0 (0.0%)	4 (1.32%)
Erythromycin	1 (0.7%)	0 (0.0%)	1 (0.3%)
Metronidazole	0 (0.0%)	2 (1.2%)	2 (0.7%)
Minocycline Hydrochloride	0 (0.0%)	1 (0.6%)	1 (0.3%)
Piperacillin Sodium · Tazobactam Sodium	3 (2.1%)	0 (0.0%)	3 (1.0%)
Rifampicin	3 (2.1%)	3 (1.8%)	6 (2.0%)
Sulfamethoxazole	1 (0.7%)	0 (0.0%)	1 (0.3%)
Trimethoprim	1 (0.7%)	0 (0.0%)	1 (0.3%)
Vancomycin Hydrochloride	30 (21.3%)	11 (6.7%)	41 (14.5%)
**Analgesics**	**8 (5.7%)**	**9 (5.5%)**	**17 (5.6%)**
Aceclofenac	2 (1.4%)	0 (0.0%)	2 (0.7%)
Acetaminophen	0 (0.0%)	1 (0.6%)	1 (0.3%)
Aspirin	1 (0.7%)	0 (0.0%)	1 (0.3 %)
Dexibuprofen	0 (0.0%)	1 (0.6%)	1 (0.3%)
Ibuprofen	0 (0.0%)	2 (1.2%)	2 (0.7%)
Ketorolac Tromethamine	2 (1.4%)	0 (0.0%)	2 (0.7%)
Loxoprofen Sodium	2 (1.4%)	0 (0.0%)	2 (0.7%)
Meloxicam	0 (0.0%)	4 (2.5%)	4 (1.32%)
Naproxen	0 (0.0%)	1 (0.6%)	1 (0.3%)
Pethidine Hydrochloride	1 (0.7%)	0 (0.0%)	1 (0.3%)
**Antifungal/Antiviral**	**5 (3.5%)**	**1 (0.6%)**	**6 (2.0%)**
Adefovir Dipivoxil	2 (1.4%)	0 (0.0%)	2 (0.7%)
Amphotericin B	2 (1.4%)	0 (0.0%)	2 (0.7%)
Entecavir	1 (0.7%)	0 (0.0%)	1 (0.3%)
Famciclovir	0 (0.0%)	1 (0.6%)	1 (0.3%)
**Chemotherapy**	**5 (3.5%)**	**13 (8.0%)**	**18 (5.9%)**
Carboplatin	0 (0.0%)	1 (0.6%)	1 (0.3%)
Cisplatin	3 (2.1%)	3 (1.8%)	6 (2.0%)
Cyclophosphamide	1 (0.7%)	0 (0.0%)	1 (0.3%)
Doxorubicin Hydrochloride	0 (0.0%)	2 (1.2%)	2 (0.7%)
Oxaliplatin	0 (0.0%)	3 (1.8%)	3 (1.0%)
Vincristine Sulfate	1 (0.7%)	4 (2.5%)	5 (1.6%)
**Immunosuppressants**	**37 (26.2%)**	**42 (25.8%)**	**79 (26.0%)**
Cyclosporine	1 (0.7%)	14 (8.6 %)	15 (4.9%)
Everolimus	0 (0.0%)	5 (3.1%)	5 (1.6%)
Mycophenolate Mofetil	3 (2.1%)	7 (4.3%)	10 (3.3%)
Tacrolimus Hydrate	33 (23.4%)	16 (9.8%)	49 (16.1%)
**ACEi/ARB/Spironolactone**	**6 (4.3%)**	**0 (0%)**	**6 (2.0%)**
Candesartan Cilexetil	1 (0.7%)	0 (0.0%)	1 (0.3%)
Enalapril Maleate	1 (0.7%)	0 (0.0%)	1 (0.3%)
Irbesartan	1 (0.7%)	0 (0.0%)	1 (0.3%)
Spironolactone	3 (2.1%)	0 (0.0%)	3 (1.0%)
**Contrast agents**	**1 (0.7%)**	**2 (1.2%)**	**3 (1.0%)**
Iodixanol	0 (0.0%)	1 (0.6%)	1 (0.3%)
Iohexol	1 (0.7%)	1 (0.6%)	2 (0.7%)
**Glucocorticoids**	**1 (0.7%)**	**72 (44.2%)**	**73 (24.0%)**
Dexamethasone	0 (0.0%)	71 (43.6%)	71 (23.4%)
Prednisolone	1 (0.7%)	1 (0.6%)	2 (0.7%)
**Diuretics**	**5 (3.5%)**	**0 (0%)**	**5 (1.6%)**
Furosemide	3 (2.1%)	0 (0.0%)	3 (1.0%)
Hydrochlorothiazide	2 (1.4%)	0 (0.0%)	2 (0.7%)
**ETC**	**23 (16.3%)**	**5 (3.1%)**	**28 (9.2%)**

This table elaborates the number of non-SAE and SAE ADE cases per medication and medication group. **Abbreviations:** ACEi: angiotensin converting enzyme inhibitor; ARB: angiotensin receptor blocker.

**Table 4:** Association between drug class and seriousness of nephrotoxicity.

	WHO-ATC Code	ROR	95% CI	*p*-Value
Antibiotics/Antimycobacterial	J01/J04	0.24	0.133–0.433	<0.001
Rifampin		0.862	0.171–4.343	0.815
Vancomycin		0.268	0.129–0.557	<0.001
**Analgesics**	**M01**	**0.972**	**0.365–2.589**	**0.847**
NSAIDs		1.161	0.393–3.431	0.997
**Chemotherapy**	**L01**	**2.357**	**0.819–6.785**	**0.166**
Cisplatin		0.862	0.171–4.343	0.815
Vincristine		3.522	0.389–31.883	0.46
**Immunosuppressants**	**L04**	**1.484**	**0.891–2.473**	**0.165**
Mycophenolate mofetil		2.064	0.524–8.137	0.464
Tacrolimus		0.356	0.187–0.680	0.002

This table elaborates the risk of reporting SAEs per each medication and medication class. ROR was estimated by the disproportionality analysis. **Abbreviation:** WHO-ATC: World Health Organization-Anatomical Therapeutic Chemical Classification System; ROR: reporting odds ratio; CI: confidence interval.

**Table 5:** Factors associated with drug-induced nephrotoxicity.

	Predictors	OR	95% CI	*p*-Value
Interstitial Nephritis	Men	1 (reference)		0.300
	Women	1.440	0.723–2.873	
	**Aging**	**1.019**	**1.001–1.038**	**0.041**
	**Polypharmacy**	**4.190**	**1.768–9.926**	**0.001**
Toxic Nephrotoxicity	Men	1 (reference)		0.845
	Women	1.0598	0.593–1.895	
	Aging	1.002	0.988–1.016	0.765
	**Polypharmacy**	**0.0543**	**0.007–0.408**	**0.005**
Overall SAE	Men	1 (reference)		0.002
	**Women**	**2.700**	**1.450–5.008**	
	**Aging**	**0.955**	**0.940–0.972**	**<0.001**
	Polypharmacy	0.726	0.293–1.798	0.621
Hospitalization SAE	Men	1 (reference)		0.139
	Women	1.663	0.848–3.261	
	**Aging**	**0.968**	**0.952–0.984**	**<0.001**
	Polypharmacy	2.087	0.863–5.047	0.102

This table elaborates the predictors that have clinical significance of increasing the risk of interstitial nephritis, toxic nephropathy, overall SAE, and hospitalization SAEs. The OR was estimated with multivariate logistic regression. **Abbreviation:** OR: odds ratio; SAE: serious adverse event.

## Data Availability

The data underlying this article cannot be shared publicly due to the ethical and privacy policies of the Korean Institute of Drug Safety & Risk Management (Ministry of Food and Drug Safety). The data will be shared upon reasonable request to the corresponding author.
